# Immunotherapy for *ALK*-Rearranged Non-Small Cell Lung Cancer: Challenges Inform Promising Approaches

**DOI:** 10.3390/cancers13061476

**Published:** 2021-03-23

**Authors:** Kamya Sankar, Sunitha Nagrath, Nithya Ramnath

**Affiliations:** 1 Division of Hematology and Oncology, Department of Internal Medicine, University of Michigan, Ann Arbor, MI 48109-5848, USA; ksankar@med.umich.edu; 2 Department of Chemical Engineering, University of Michigan, Ann Arbor, MI 48109-5848, USA; snagrath@umich.edu; 3 Rogel Cancer Center, University of Michigan, Ann Arbor, MI 48109-5848, USA; 4 Division of Medical Oncology, Veterans Affairs Ann Arbor Healthcare System, Ann Arbor, MI 48109-5848, USA

**Keywords:** *ALK*, *ALK*-rearrangements, non-small cell lung cancer, tyrosine kinase inhibitors, immune evasion, immunotherapy, cancer vaccine

## Abstract

**Simple Summary:**

Non-small cell lung cancer is the most common type of lung cancer. Anaplastic Lymphoma Kinase (*ALK*) rearrangements have been found in 5–6% of non-small cell lung cancers. While *ALK* rearranged non-small cell lung cancer is exquisitely sensitive to *ALK* directed targeted therapies, it is generally resistant to immune-based therapies. We aim to describe the mechanisms by which *ALK*-rearranged non-small cell lung cancers escape host immunity and are thereby unresponsive to immunotherapies. Furthermore, we describe new immunotherapy strategies and the promises and challenges in incorporating these into clinical practice.

**Abstract:**

Rearrangements in the Anaplastic Lymphoma Kinase (*ALK*) gene have been implicated in 5–6% of all non-small cell lung cancers. *ALK*-rearranged non-small cell lung cancers are sensitive to *ALK*-directed tyrosine kinase inhibitors, but generally resistant to single-agent immune checkpoint inhibitors. Here, we aim to describe the mechanisms of *ALK* aberrations in non-small cell lung cancer by which an immunosuppressed tumor microenvironment is created, leading to host immune evasion. We report pre-clinical and clinical studies evaluating novel immunotherapeutic approaches and describe the promises and challenges of incorporating immune-based treatments for *ALK*-rearranged non-small cell lung cancer.

## 1. Introduction

Non-small cell lung cancer (NSCLC) accounts for 80% of lung cancers and is one of the leading causes of cancer-related mortality worldwide [1,2]. In 2007, NSCLC tumors harboring mutations in the anaplastic lymphoma kinase (*ALK*) gene were identified [3]. *ALK* rearrangements result in an oncogenic driver in 5–6% of NSCLC cases [4], representing approximately 100,000 new cases of lung cancer annually worldwide. Patients with *ALK*-rearranged NSCLC are characterized by an absence of smoking or light smoking history and a younger age at diagnosis (median age at diagnosis: 54 years) [5].

### 1.1. ALK Oncogene

The *ALK* gene encodes a tyrosine kinase receptor which shares a high level of similarity with the insulin receptor but has a unique glycine-rich extracellular domain. The intracellular domain of the *ALK* protein shares 80% homology with leukocyte tyrosine kinase (LTK), which has been implicated in immune-related and inflammatory diseases. The human *ALK*/LTK family is activated by small peptide ligands, FAM150A(AUGβ) and FAM150B(AUGα) [6,7]. The physiologic role of wildtype *ALK* is yet to be understand completely, but tissue expression has been found to be restricted to the peripheral and central nervous systems [8]. These are considered to be “immune-privileged sites”, i.e., sites with sufficient immune tolerance to self-antigens [9]. Thus, the *ALK* protein may represent a potential antigen for the human immune system [10,11].

*ALK* was first discovered to play a role in oncogenesis in 1994 when the *NPM-ALK* fusion was identified in anaplastic large-cell non-Hodgkin’s lymphoma (ALCL) [12]. Since then, fusions, mutations, and alternative splicing of *ALK* has been detected in several cancers including NSCLC, renal cell carcinoma, thyroid cancer, digestive tract cancer, breast cancer, ovarian carcinoma and leukemia [13,14,15]. The *EML4-ALK* fusion was first observed in five out of 75 (6.7%) Japanese patients with lung cancer, occurring mutually exclusive of other driver mutations such as *EGFR* or *KRAS* [3]. *EML4-ALK* translocation results in constitutive *ALK* tyrosine kinase activity, representing an oncogenic addiction pathway in lung cancer. The frequency of *EML4-ALK* fusion in NSCLC has ranged from 2% to 7% in several studies, with an average frequency of ~5% [16,17]. Moreover, 19 other fusion partners have been identified in *ALK*-rearranged NSCLC including *KIF5B, TGF, TPR,* and *KLC1* [18,19].

### 1.2. Models and Techniques to Study ALK-Rearranged NSCLC In Vivo

The *EML4*-*ALK* fusion gene was first identified in a mouse model in which inversion within chromosome 2p resulted in an *EML4-ALK* fusion gene. Forced expression of *EML4-ALK* in mouse 3T3 fibroblasts resulted in subcutaneous tumors in nude mice [3], while oral administration of an *ALK*-directed tyrosine kinase inhibitor (TKI) resulted in rapid tumor regression [20]. However, drug screening and modeling for *EML4-ALK* positive NSCLC cells in vitro and in in vivo mouse models has been challenging. Newer models have used viral-mediated delivery of the CRISPR/cas9 system to somatic cells of adult animals to induce chromosomal rearrangements. A CRISPR/cas9 adenoviral vector was used to generate a mouse model of *EML4-ALK* positive lung cancer [21]. These tumors displayed typical molecular features of *ALK*-rearranged human NSCLC and responded to treatment with *ALK* TKI. Similarly, a CRISPR/cas9 lentiviral vector was used to generate *EML4*-*ALK* positive lung cancers in mice models via intratracheal or intrapulmonary inoculation [22]. The CRISPR/cas9 system provides unique opportunities to test the efficacy of targeted therapies, investigate mechanisms of drug resistance, and to test newer immune-based therapeutic strategies for *ALK* rearranged lung cancer in preclinical in vivo models.

### 1.3. Targeted Therapy for ALK-Rearranged NSCLC

Targeted therapies with small-molecule TKIs have revolutionized the prognosis and management of oncogene-addicted NSCLC. Crizotinib, a multi-targeted TKI with activity against *MET, ALK*, and *ROS1*, was the first *ALK* inhibitor to be approved by the U.S. Food and Drug Administration in 2011 for previously untreated metastatic *ALK*-rearranged NSCLC, based on results of a single-arm study that demonstrated an objective response rate of 61% [23]. Since then, four additional TKIs have been approved for treatment of advanced *ALK*-rearranged NSCLC including ceritinib, alectinib, brigatinib, and lorlatinib. Patients with *ALK*-rearranged NSCLC generally can receive 2–3 *ALK* TKIs sequentially prior to moving on to a non-targeted systemic treatment at the time of progression.

While targeted therapies have led to significant improvement in the management and prognosis of oncogene-addicted NSCLC, acquired resistance to TKIs is almost always inevitable. Resistance to *ALK* inhibitors can be mediated by point mutations in the *ALK* kinase domain, amplifications in the *ALK* gene or constitutive activation of bypass oncogenic pathways (i.e., EGFR, c-KIT, c-MET, and IGF-R1) [24,25,26]. Further understanding of bypass resistance mechanisms to *ALK* inhibitors and exploration of other therapeutic strategies, for example with immune-based or combination approaches, is required to prolong duration of treatment response and offer additional treatment options.

### 1.4. The Current Landscape of Immunotherapeutic Approaches for Treatment of ALK-Rearranged NSCLC

Immune checkpoint inhibitors (ICI) targeting programmed cell death-1 (PD-1), programmed cell death ligand-1 (PD-L1) and CTLA-4 are promising tools against NSCLC. However, these therapies are less effective in those harboring oncogenic driver mutations, such as *EGFR* or *ALK*. Pre-clinical and clinical studies have demonstrated the up-regulation of immune checkpoints such as PD-L1 in de novo *EGFR*-mutant and *ALK*-rearranged tumors [27,28,29,30] leading to the question of whether ICI alone or in combination with targeted TKI would offer clinical benefit in *ALK*-altered NSCLC. However, randomized controlled trials evaluating ICI in NSCLC either have not included patients with *ALK*-rearrangements or have not had sufficient numbers of patients to provide meaningful subgroup analysis [31,32,33,34]. Retrospective studies from a global multicenter network of patients and a national database of U.S. oncology practices have reported patients with *ALK*-rearranged NSCLC tumors to be relatively refractory to single-agent ICI therapy (median progression-free survival of ~2–3 months) [11,35], indicating that the clinical benefit of ICI in *ALK*-rearranged NSCLC is modest to poor. Herein, we aim to discuss the immune biology of *ALK*-rearranged NSCLC, mechanisms which lead to immune resistance, and existing pre-clinical and clinical data regarding immunotherapeutic approaches for treatment of *ALK*-rearranged NSCLC which may inform novel treatment strategies in the future.

## 2. Tumor Microenvironment of ALK-Rearranged NSCLC

### 2.1. The Immunosuppressive Microenvironment in ALK-Rearranged NSCLC

**Figure 1:**
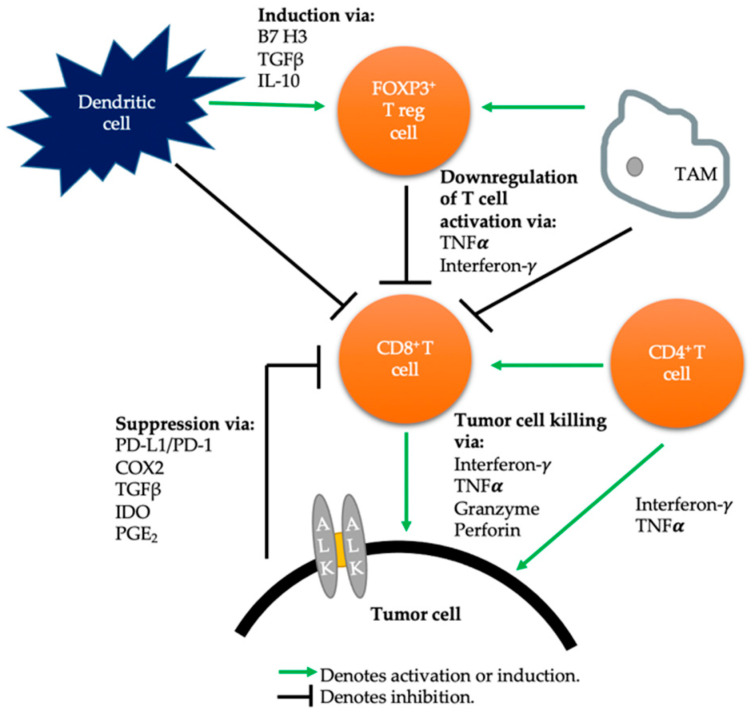
Tumor microenvironment in *ALK*-wildtype non-small cell lung cancer. TGFβ: Transforming Growth Factor β. IL-10: Interleukin 10. TNFα: Tumor necrosis factor alpha. IDO: Indoleamine 2,3-dioxygenase. PGE_2_: Prostaglandin E2. COX2: Cyclooxygenase 2.

PD-L1 expression is one of the primary immunosuppressive drivers in many cancers, but immune evasion is facilitated by various cells of the immune system [36]. In 2004, the concept of immunoediting was described to explain the relationship between the tumor and its microenvironment, involving an elimination phase, equilibrium phase, and the immune evasion phase [37] (Figure 1: Tumor microenvironment in NSCLC with wild type *ALK*).

The tumor microenvironment (TME) in *ALK*-rearranged caners includes interaction of tumor-associated macrophages (TAMs), CD4^+^ T cells, CD8^+^ T cells, regulatory T cells (FOXP3^+^) and mast cells [38]. In ALCL, pre-clinical models have shown a positive correlation between PD-L1 expression in the tumor cells and TAMs in *ALK*-positive ALCL and conversely, a negative correlation in *ALK*-negative ALCL [39,40]. The ultimate fate of the relationship between PD-L1 expression and FOXP3^+^ T regulatory cells is to rescind effector CD8^+^ T cell-mediated tumor response ultimately leading to T cell exhaustion [41]. Furthermore, PD-L1 expression has been shown to play an important role in regulating induced T regulatory cells and sustaining their function [42]. The ratio of CD8^+^:FOXP3^+^ TILs has been shown to be more informative than each being assessed separately, as the ratio more accurately represents the reciprocal interplay between the effector CD8^+^ TILs and repressive FOXP3^+^ T regulatory cells in the TME [43].

### 2.2. Pre-Clinical Studies Evaluating the Role of PD-1/PD-L1 in Immune Evasion of ALK-Rearranged NSCLC

PD-L1 and PD-L2 are commonly overexpressed on tumor cell surfaces [44,45]. Forced expression of PD-L1 on the surface of tumor cells in mice inhibits T-cell mediated anti-tumoral immune responses via the PD-1/PD-L1 pathway [46,47]. Upregulation of the PD-1 pathway may be mediated by innate and adaptive resistance mechanisms [48]. Constitutive upregulation of PD-L1 is an example of innate resistance, as was shown in ALCL where *NPM-ALK* induces CD274 (PD-L1) expression by activating transcription factor STAT3. Binding of STAT3 to the CD274 gene promoter in vitro and in vivo was required for PD-L1 gene expression [30]. In contrast, adaptive immune resistance may induce PD-L1 expression in response to local inflammatory signals produced by an active anti-tumor response.

**Figure 2:**
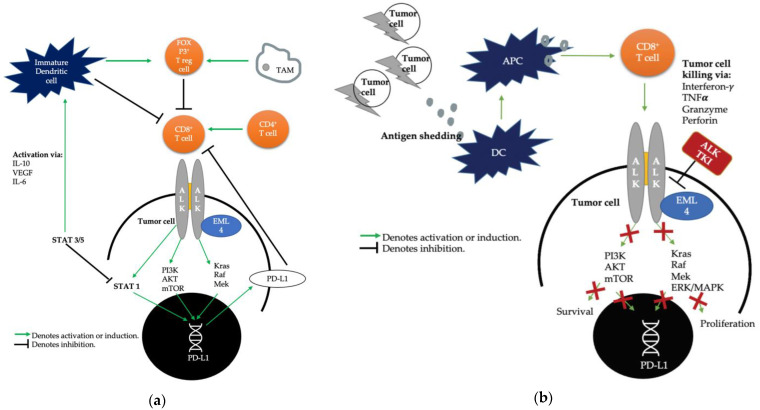
(**a**) Tumor microenvironment in *ALK*-rearranged non-small cell lung cancer. (**b**) Tumor microenvironment in *ALK*-rearranged non-small cell lung cancer in presence of *ALK* TKI. IL-10: Interleukin 10. IL-6: Interleukin 6. STAT3/5: Signal transducer and activator of transcription 3 and 5. PI3K: Phosphoinositide 3-kinase. AKT: protein kinase B. mTOR: mechanistic target of rapamycin. Kras: Kirsten rat sarcoma. Raf: Rapidly accelerated fibrosarcoma. Mek: mitogen-activated protein kinase. ERK/MAPK: extracellular signal-regulated kinase/mitogen-activated protein kinase. APC: antigen-presenting cell. DC: Dendritic cell.

In NSCLC tumors, the *EML4-ALK* fusion is hypothesized to create an immunosuppressive TME by activation of downstream oncogenic signaling pathways leading to the activation of the PI3K, MAPK, and Hippo pathways [29,49] (Figure 2a). Koh et al. were the first to report the correlation of *EML4-ALK* and increased PD-L1 expression through regulation of transcription factors in lung adenocarcinoma cells. Moreover, PD-L1 expression was increased by regulating hypoxia-inducible factor-1α and STAT3 activities under hypoxia, but STAT3 alone under normoxia, suggesting a differential regulation of PD-L1 expression depending on normoxia versus hypoxia in the TME [50]. Upon clinicopathological correlation of PD-L1 expression to outcomes in patients with *ALK*-rearranged NSCLC, PD-L1 expression had no influence on disease-free survival among patients who underwent surgical resection. By contrast, in 90 patients with *ALK*-rearranged adenocarcinoma who received crizotinib, strong PD-L1 expression was significantly associated with an unfavorable clinical outcome with shorter progression-free survival and overall survival. Importantly, PD-L1 was consistently retained or increased after acquired resistance to crizotinib in a subset of patients. PD-L1 expression is generally downregulated during the efficacious phase of TKI treatment (Figure 2b). Therefore, up-regulation of PD-L1 at time of acquired resistance to TKI may be due to the restoration of *ALK* in settings of failed *ALK* inhibition leading to *ALK*-mediated upregulation of PD-L1 or may represent a PD-1/PD-L1 pathway mediated immune escape mechanism [50].

Upregulation of PD-L1 expression in *ALK*-rearranged NSCLC was supported by an in vitro study by Hong et al., where cell apoptosis and viability tests were used to study immune suppression by *ALK* activation and immune reactivation by *ALK* TKIs and anti-PD-1 antibody in a NSCLC tumor cell and DC-CIK cell co-culture system. Overexpression of *ALK* fusion protein increased PD-L1 expression, leading to increased apoptosis of T cells. While *ALK* TKIs directly inhibited tumor viability and enhanced antitumor immunity via downregulation of PD-L1, anti-PD-1 antibody was effective in both crizotinib sensitive and resistant cell lines, suggesting the need for further study in anti-PD-1 blockade for treatment of *ALK*-rearranged NSCLC [28]. Similar trends were noted by Ma et al. in a study evaluating NSCLC cell lines concurrently with patient samples. PD-L1 expression was higher in cell lines harboring the *EML4-ALK* fusion gene via expression of downstream pathways such as AKT, ERK, and STAT3, while incubating with crizotinib led to significant downregulation of PD-L1. The activity of crizotinib-sensitive cells was also inhibited by a PD-1 targeted agent and combination of both drugs inhibited cell viability more strongly than with crizotinib alone [51]. These findings were supported by another study evaluating 134 surgically resected NSCLC specimens which found PD-L1 expression by immunohistochemistry to be higher in the cell lines positive for *EML4-ALK* compared to those negative for the fusion gene. The forced expression of *EML4-ALK* markedly increased PD-L1 expression in adenocarcinoma cells. Endogenous expression of PD-L1 in *EML4-ALK* positive NSCLC was attenuated by treatment with *ALK* inhibitor alectinib as well as inhibitors of downstream pathways (MEK-ERK and PI3K-AKT) [29]. The aforementioned studies imply a direct link between the constitutive oncogene pathway activation by *ALK* and upregulation of PD-L1 expression as an adaptive mechanism for immune evasion in *ALK*-rearranged NSCLC.

### 2.3. Clinical Studies Correlating PD-L1 Expression and Response to Immune Checkpoint Inhibitors in Patients with ALK-Rearranged NSCLC

Despite preclinical studies correlating *ALK*-rearrangement with upregulation of PD-L1 expression, clinical studies have not confirmed this. In fact, the opposite has been suggested by retrospective studies where *ALK*-rearranged NSCLC has correlated with low PD-L1 expression and poor response to ICI. In a retrospective analysis of 58 patients receiving anti-PD-1 ICI for advanced NSCLC, a subset of patients with *ALK*-rearrangement had low PD-L1 expression, low CD8^+^ TILs, and low response rates to anti-PD1 blockade [52]. Another study with 715 NSCLC patients had similar findings, where a subset analysis of patients with *EGFR* or *ALK* rearrangements had the lowest proportion of PD-L1(+)/CD8^+^(+) tumors and highest proportion of PD-L1(−)/CD8^+^(−) tumors. Furthermore, patients with PD-L1 positive tumors had the shortest median overall survival [53]. A recent retrospective analysis of East Asians showed similar findings, where patients with *EGFR*-mutant or *ALK*-rearranged NSCLC had the highest proportion of PD-L1(−)/CD8^+^(−) tumors. Gene expression profiling of 11 *ALK*-rearranged NSCLC tumors was performed and showed an immune infiltrating phenotype characterized by a lack of CD8^+^ T cells or activated memory CD4^+^ T cells [54].

Upregulation of PD-L1 expression in *ALK*-rearranged NSCLC as shown in preclinical models has not translated to an improved clinical benefit with ICI. This may be due to multiple factors, including a TME that is characterized by lack of infiltrating CD8^+^ T cells and complex interplay of constituents such as PD-L1 expressing TAMs and induced FOXP3^+^ T regulatory cells. Furthermore, *ALK*-rearranged NSCLC present with fewer non-synonymous mutations compared with smoking associated NSCLC [55], that result in a less inflamed TME as well as less neo-antigen generation, leading to a potentially weaker response to ICI.

## 3. Immunotherapy Approaches in Treatment of ALK-Rearranged NSCLC

### 3.1. The Role of PD-1 Axis Checkpoint Inhibitors in ALK-Rearranged NSCLC

The majority of prospective randomized controlled trials evaluating efficacy of ICI in advanced NSCLC have excluded those with *ALK*-rearrangements or have had insufficient numbers of patients to provide meaningful subgroup analyses.

#### 3.1.1. Retrospective Studies Evaluating ICIs in ALK-Rearranged NSCLC

Retrospective studies have shown lack of efficacy with single-agent ICI in *ALK*-rearranged NSCLC. In a single-institutional retrospective study evaluating patients with NSCLC who were treated with ICI between 2011 and 2016, none of the *ALK*-positive patients had an objective response, in comparison with an objective response of 23.3% in *ALK*-negative patients [52]. In another multicenter retrospective analysis, among 23 *ALK*-rearranged NSCLC patients, none had a confirmed radiographic response while 21% had stable disease and 78% progressed [35]. In a global multicenter cancer registry database, Jahanzeb et al. reported similarly low response rates in 83 patients with *ALK*-rearranged NSCLC receiving ICI, where median time to discontinuation of ICI was 2.1 months and median progression-free survival was 2.3 months [11]. These findings are summarized in Table 1.

#### 3.1.2. Prospective Clinical Trials Evaluating ALK-Rearranged NSCLC Patients Treated with ICIs

To our knowledge, the only prospective randomized clinical trials evaluating efficacy of ICIs in NSCLC which have included *EGFR*-mutant or *ALK*-rearranged NSCLC patients are the following: CheckMate 057, KEYNOTE-010, POPLAR, and OAK (Table 2). In CheckMate 057, nivolumab was compared to docetaxel in patients with advanced NSCLC who had progressed on doublet chemotherapy. Thereby, 82 of 292 (14%) patients in the nivolumab group and eight of 290 (3%) patients in the docetaxel group were *ALK*-positive. The *EGFR*-mutant patients in the nivolumab group had no survival benefit, and responses in the *ALK*-rearranged group were not reported due to too few patients [32]. KEYNOTE-010 evaluated the efficacy of pembrolizumab versus docetaxel in previously treated advanced NSCLC patients. Two of 344 patients in the pembrolizumab 2 mg/kg group, four of 346 patients in the pembrolizumab 10 mg/kg group and 2 of 342 patients in the docetaxel group harbored *ALK*-rearrangements. Similar to CheckMate 057, the *EGFR*-mutant patients did not have a survival benefit with pembrolizumab, and outcomes for the *ALK*-positive patients were not reported due to too few patients [33]. In both the POPLAR and OAK trials, though *ALK*-rearranged patients were allowed, these were only represented in the chemotherapy groups [34]. These findings are summarized in Table 2.

### 3.2. The Role of PD-1 Axis Inhibitors in Combination with ALK-directed TKI

Combination strategies of *ALK* TKI with ICI therapy have been studied and preliminary data has shown activity. However, long-term efficacy outcomes are yet to be reported and toxicity rates are non-trivial. CheckMate 370 was a phase I/II study of the combination of nivolumab plus crizotinib in previously untreated advanced *ALK*-rearranged NSCLC. Of the first 13 patients treated, five (38%) developed severe hepatic toxicity leading to discontinuation of the treatment, and two (15%) died of treatment-related adverse effect leading to discontinuation of patient enrollment [56]. In another phase IB dose-escalation trial of 36 previously treated or TKI-naïve *ALK*-rearranged NSCLC patients, nivolumab 3 mg/kg every two weeks was evaluated in combination with ceritinib given 450 mg/day or 300 mg/day. Among TKI-naïve patients, the objective response rate was 83% in the ceritinib 450 mg/day cohort versus 70% in the ceritinib 300 mg/day cohort and PD-L1 positivity correlated with higher response rates. However, frequent grade 3 or 4 adverse events including increases in liver function enzymes, rash and diarrhea, required protocol modification to address toxicities and ultimately enrollment discontinuation [57]. JAVELIN 101 is a phase IB/II clinical trial evaluating previously treated *ALK*-rearranged NSCLC patients with either avelumab plus lorlatinib or avelumab plus crizotinib. Preliminary data showed objective response rate of avelumab plus lorlatinib of 46.7% versus avelumab plus crizotinib of 16.7%, along with an acceptable safety profile in the avelumab plus lorlatinib group [58] (NCT02584634). Ipilimumab, a CTLA4 inhibitor, was also studied in combination with erlotinib or crizotinib for *EGFR*-mutated or *ALK*-rearranged NSCLC patients in a phase I trial, where a median of four doses of ipilimumab were given to the entire cohort. Of three *ALK*-rearranged patients, one developed hypophysitis requiring treatment discontinuation and one developed grade 2 pneumonitis, with a median PFS reported of 24.1 months. Other studies evaluating similar combination strategies have been reviewed elsewhere [59] though still require long term outcomes data to assess safety and efficacy. The trials involving *ALK* TKI with ICI have not yet been evaluated in a randomized phase II or III clinical trial. However, the preceding data suggest that this combination strategy in general has a poor therapeutic ratio with modest benefit and a high toxicity profile.

### 3.3. Adoptive T cell Approaches in Treatment of ALK-Rearranged NSCLC

Though chimeric antigen receptor (CAR) T cell therapy has been effective in treatment of hematologic malignancies, treating solid tumors using CAR-T cell therapy remains a significant challenge. A pilot study using anti-MUC1 CAR-T cells with PD-1 knockout to treat advanced NSCLC patients showed an objective response in two out of six evaluable patients though the therapeutic benefit was variable in each patient. A phase I study (NCT03330834) evaluating PD-L1 CAR-T cells in patients with advanced lung cancer terminated early due to a serious adverse event in the one enrolled participant.

CAR-T approaches are enticing particularly for tumors with immunosuppressed TMEs, such as *ALK*-rearranged NSCLC, however, there are several challenges to overcome including improvement in the CAR structure, identification of specific tumor antigens to target, overcoming complexities of the TME, and accessing/penetrating large volume solid tumors for effective treatment [60]. CAR-based immunotherapy has previously been studied in *ALK*-positive pediatric solid tumors. An *ALK*-directed CAR incorporating the single-chain variable fragment sequence of the *ALK*48 monoclonal antibody produced a local cytokine-mediated inflammatory response and demonstrated cytotoxic activity of *ALK*-expressing tumor cells in vitro but had finite anti-tumor activity in xenograft models of neuroblastoma [61]. CAR-T induced anti-tumor activity was found to be highly dependent on the density of target antigens and CAR density on T cells.

Recently, adoptive cell therapy, a technique that utilizes ex vivo expanded T lymphocytes to increase the effector cell pool in tumors, has been suggested as a therapeutic approach in NSCLC. NSCLC has one of the highest clonal mutational burdens, providing rationale to develop clonal neoantigen reactive T cells (cNeT) for this indication [62]. Robertson et al. described a method in which autologous TILs are isolated from a sample of the patient’s tumor and clonal neoantigens are identified by whole exome and RNA sequencing. Dendritic cells are then loaded with patient-specific clonal neoantigens and cultured with TILs, producing ATL001. ATL001 is therefore derived from autologous TILs designed to target patient-specific neoantigens which are specific to tumor cells and may be absent in healthy tissue. ATL001 is being evaluated in patients with advanced NSCLC (NCT04032847) [63].

### 3.4. Cancer Vaccine Therapy in Advanced ALK-Rearranged NSCLC

The concept of vaccines for treatment of cancers is to stimulate antigen-specific immune response by presenting a tumor associated antigen (TAA) [64]. Vaccines have been used for both prevention and treatment of cancer. Relevant examples include the recombinant human papillomavirus vaccine [65] and an immunogenic personalized neoantigen vaccine (derived from TAA) for patients with melanoma [66]. For NSCLC, two vaccines are approved in other countries. The CIMAvax Epidermal Growth Factor (EGF) vaccine is a chemical conjugate of the Epidermal Growth Factor with the P64 protein from Meningitis B bacteria. The CIMAvax EGF is approved in Cuba, Peru, and Venezuela for stage III and IV NSCLC after progression on first line therapy based on results from a phase II randomized controlled study showing safety and efficacy when compared to best supportive care [67]. Patients with advanced NSCLC were randomized to EGF vaccinations or best supportive care after progression on first-line chemotherapy. A good antibody response, representative of immunogenicity of the vaccine, was detected in 51.4% of vaccinated patients. While the vaccinated group had a trend towards improved survival (median OS 12.7 vs. 8.5 months), these differences were not statistically significant. This vaccine has not yet been approved in other countries though trials are ongoing in the United States, Europe, and Japan. Racotumomab is a murine IgG1 antibody directed against NeuGcGM3 ganglioside, a tumor antigen expressed in NSCLC, and is approved in Argentina and Cuba for advanced NSCLC after progression on first line therapy [64]. This was based on a randomized placebo-controlled multicenter trial which randomized patients to racotumomab or placebo maintenance if patients had stable disease after first-line chemotherapy. The median PFS and OS was improved in the racotumomab arm with statistical significance. Racotumomab has yet to be evaluated in the United States or Europe [68]. In the United States, though several types of vaccines (allogenic, peptide, autologous, DNA, and vector-based) have demonstrated safety and good tolerability in advanced NSCLC, the clinical benefit defined in response rates and survival outcomes have been poor in clinical trials. Combination approaches are now being evaluated including vaccine in combination with ICI, vaccine in combination with chemotherapy, and vaccine in combination with ICI and chemotherapy. These include a phase I/II clinical trial with CIMAvax-EGF in combination with nivolumab or pembrolizumab (NCT02955290) (currently recruiting), a phase I/II trial testing Adenovirus Expressing MAGE-A3 and MG1-MAGEA3 in combination with pembrolizumab (NCT02879760) (closed; data yet to be reported) and a phase II trial evaluating combination of TG4010 with nivolumab (NCT02823990) (study terminated due to low enrollment).

In *ALK*-rearranged NSCLC, one strategy to convert the immunosuppressed TME to an immune-responsive TME is by use of an *ALK*-directed vaccine, in order to stimulate TILs into the TME [10]. *ALK* has been shown to be antigenic in human lymphomas, with demonstration of spontaneous B and T cell responses against the *ALK* protein by measurable antibodies, CD8^+^ cytotoxic T cells and CD4^+^ helper T cells directed against *ALK* epitopes [69,70]. Furthermore, tissue expression of *ALK* in humans is largely restricted to the central and peripheral nervous systems [8]. Due to minimal systemic exposure to *ALK* throughout human development, the *ALK* protein has many features of an ideal tumor antigen to elicit a vaccine response against. Voena et al. developed an *ALK* vaccine containing a DNA plasmid coding the intracytoplasmic domain of *ALK*, which was shown to be effective in a mouse model of *ALK*-positive NSCLC by eliciting a tumor-specific cytotoxic response and preventing tumor growth. In mice models with *EML4-ALK* NSCLC, the average number of tumors detected in control mice was 58 whereas *ALK*-vaccinated mice only had 16 at week 20. Survival was extended by 18 weeks in *EML4-ALK* mice which were vaccinated as compared to control. In examination of the TME, *ALK* vaccine was shown to increase the number of intratumoral T cells with a higher CD8^+^:CD4^+^ ratio, and higher CD8^+^: FOXP3^+^ ratio. The authors then tested the impact of the PD-1/PD-L1 pathway on the efficacy of the *ALK* vaccine. In the presence of high PD-L1 expression, the vaccine was less effective, but could be overcome by concurrent treatment with anti-PD-1 antibody [71]. Furthermore, in vaccinated mice, eventual tumor growth led to death, likely due to development of resistance mechanisms and immune evasion. Diminished efficacy over time due to upregulation of immune checkpoints such as PD-L1 could be overcome by concurrent administration of anti-PD-L1 antibodies. Another DNA plasmid peptide vaccine was also shown to be effective in *ALK*-positive NSCLC mouse models [72]. Though an *ALK* vaccine for *ALK*-positive NSCLC has yet to be tested in humans, it is currently positioned for a future phase I clinical trial [73].

## 4. Conclusions

*ALK*-rearranged NSCLC comprise 5–6% of all NSCLC and are exquisitely sensitive to *ALK*-directed TKIs, four of which are now approved for treatment of patients with advanced *ALK*-rearranged NSCLC. After failure on one or more *ALK* TKIs, salvage approaches include chemotherapy and/or chemo-immunotherapy combination strategies. Despite pre-clinical studies suggesting up-regulation of PD-L1 in *ALK*-rearranged NSCLC, single-agent ICI have not shown clinical benefit in retrospective studies in the treatment of *ALK*-rearranged NSCLC. This is thought to be due to an *ALK* driven immunosuppressive TME characterized by scarce CD8+ TIL and increased FOXP3+ T regulatory cells [74]. The ENIGMA+ trial, a biorepository of *ALK*-rearranged NSCLC patients in order to further understand the interactions between the tumor and host immunity, is now open to researchers in this field in order to foster development of effective immune-based treatment approaches for *ALK*-rearranged NSCLC [75]. We have summarized existing data regarding novel immunotherapeutic strategies which may be considered for *ALK*-rearranged NSCLC. The data from phase I/II clinical trials evaluating *ALK* TKI in combination with ICI have been underwhelming, with a significant toxicity profile and a low therapeutic index. The efficacy of adoptive T cell approaches, including CAR-T cell therapy, are limited by need for identification of specific tumor antigens at sufficient densities and overcoming the complexities of the TME with an appropriate CAR structure. *ALK*-directed vaccine has shown promising efficacy in preclinical models by converting the immunosuppressed TME into a responsive one with potential of improving efficacy particularly when administered with combination strategies. *ALK*-directed vaccine in combination with *ALK* TKI may facilitate tumor cell death and release of tumor neoantigens in order to enhance vaccine-mediated antitumor immune response. An optimal time to administer the *ALK* vaccine might be immediately following maximal response from first line TKI treatment. Alternatively, *ALK*-vaccine in combination with anti-PD-1/PD-L1 ICI has shown efficacy in preclinical models by overcoming adaptive mechanisms of tumor immune evasion. Therefore, though clinical trials involving vaccine-based approaches will require high pre-clinical effort, the potential for durable efficacy with combination vaccine strategies warrants further investigation [71]. While *ALK* TKIs have significantly improved the prognosis of patients with advanced *ALK*-rearranged NSCLC, these cancers remain incurable. Further investigation in targeting the immunosuppressed TME and anticipated clinical trials evaluating *ALK* directed vaccines represent the future of improving outcomes for patients with *ALK*-rearranged NSCLC.

## Figures and Tables

**Table 1:** Retrospective studies evaluating patients with advanced *ALK*-rearranged non-small cell lung cancer treated with immune checkpoint inhibitors.

Study Description	Author/Year	Total No. of Patients	No. of Patients with *ALK* Rearrangements	Objective Response Rate in *ALK* Rearranged Patients	Median PFS in *ALK*-Rearranged Patients
Single institutional study of EGFR and *ALK*-mutated NSCLC patients treated with PD-1/PD-L1 inhibitors between 2011–2016	Gainor 2016 [52]	28	6 (21.4%)	0%	2.07 m. (95% CI: 1.87–2.17)
Multicenter international study evaluating patients with advanced NSCLC with at least one driver mutation treated with PD-1/PD-L1 inhibitor	Mazieres 2019 [35]	551	23 (4.2%)	0%	2.5 m. (95% CI: 1.5–3.7)
Multicenter study evaluating patients with *ALK*-rearranged NSCLC treated with anti PD-1/PD-L1 inhibitors	Jahanzeb 2020 [11]	83	83 (100%)	3.6%	2.34 m. (95% CI: 1.55–3.09)

**Table 2:** Prospective clinical trials evaluating immune checkpoint inhibitors in advanced non-small cell lung cancer patients which included patients with *ALK*-rearranged non-small cell lung cancer.

Trial	Author/Year	Total No. of Patients	Treatment	No. of Patients with *ALK* Rearrangements in Immunotherapy Arm vs. Chemotherapy Arm (%)	Median OS (in the Entire Study Population) *
CheckMate 057	Borghei 2015 [32]	582	Nivolumab vs. docetaxel	13 (4%) vs. 8 (3%)	12.2 vs. 9.4 m.
Keynote-010	Herbst 2016 [33]	1034	Pembrolizumab vs. docetaxel	7 (1%) vs. 2 (0.5%)	10.4 vs. 12.7 m.
POPLAR	Fehrenbacher 2016 [34]	287	Atezolizumab vs. docetaxel	0 vs. 3 (2%)	12.6 vs. 9.7 m.
OAK	Rittmeyer 2017 [34]	1225	Atezolizumab vs. docetaxel	2 (<1%) vs. 0	13.8 vs. 9.6 m.

* Median OS in the *ALK* rearranged population was not reported due to too few patients.
